# Spatially integrated cortico-subcortical tracing data for analyses of rodent brain topographical organization

**DOI:** 10.1038/s41597-024-04060-y

**Published:** 2024-11-12

**Authors:** Martin Øvsthus, Maaike M. H. van Swieten, Maja A. Puchades, Chiara Tocco, Michèle Studer, Jan G. Bjaalie, Trygve B. Leergaard

**Affiliations:** 1 Department of Molecular Medicine, Institute of Basic Medical Sciences, University of Oslo, Oslo, Norway; 2 Université Côte d’Azur, CNRS, Inserm, iBV, Nice, France

**Keywords:** Neural circuits, Neuroscience

## Abstract

The cerebral cortex extends axonal projections to several subcortical brain regions, including the striatum, thalamus, superior colliculus, and pontine nuclei. Experimental tract-tracing studies have shown that these subcortical projections are topographically organized, reflecting the spatial organization of sensory surfaces and body parts. Several public collections of mouse- and rat- brain tract-tracing data are available, with the Allen mouse brain connectivity atlas being most prominent. There, a large body of image data can be inspected, but it is difficult to combine data from different experiments and compare spatial distribution patterns. To enable co-visualization and comparison of topographical organization in mouse brain cortico-subcortical projections across experiments, we represent axonal labelling data as point data in a common 3D brain atlas space. We here present a collection of point-cloud data representing spatial distribution of corticostriatal, corticothalamic, corticotectal, and corticopontine projections in mice and exemplify how these spatially integrated point data can be used as references for experimental investigations of topographic organization in transgenic mice, and for cross-species comparison with corticopontine projections in rats.

## Background & Summary

Neuronal networks are assemblies of widely distributed, interconnected neurons contributing to different functions of the nervous system. The spatial organization of axonal projections among brain regions defines how signals may be distributed and influence neuronal networks^1–3^. A hallmark of the mammalian brain is that axonal projections are topographically organized, typically with neighbouring relations reflecting sensory surfaces, such as the skin, retina, or cochlea, or the layout of body musculature. Such spatial organization patterns are to a variable degree preserved across connected brain regions. In subcortical networks complex distribution patterns may serve to introduce new neighbouring relationships and different possibilities for cellular interactions and signal integration^4,5^.The preservation or modification of such spatial relationships has been extensively studied in the somatotopic mapping of somatosensory and somatomotor systems^6^, the retinotopic organization in the visual system^7,8^, and the tonotopic organization in the auditory system^9,10^.

Topographical organization of neuronal connections is typically investigated experimentally in small animal models by applying axonal tracer substances to discrete brain locations, to label neurons and/or axonal trajectories with branching patterns *via* retrograde or anterograde transport along the axons^11,12^. While experimental tract tracing studies traditionally have focused on mapping neural connections in one or few regions of interest, data on how distributed neural populations are interconnected and organized are needed to understand the local and global information processing in the brain^13,14^.

In response to the need for brain-wide tract tracing data^15^, large collections of tract tracing data from the mouse brain have been gathered and publicly shared^16–18^. The largest collection of such tract-tracing data is currently offered by the Allen mouse brain connectivity atlas^16,19^, comprising a catalogue of volumetric image data that can be viewed online, or downloaded as raw or segmented images^18^. The tract-tracing data are spatially integrated in a common reference atlas framework^16,20,21^ and have been widely used in a range of studies describing connections among brain regions^16,19,22–28^, as well as for a range of computational modelling efforts^29–32^. However, to discover complex patterns of topographical organization it is necessary to compare the spatial distribution of labelling across animals with tracers placed in different locations. Complex patterns of topographic organization may require three-dimensional (3D) analyses based on computerized reconstructions of labelled neuronal elements, since certain patterns may not be visible from the cardinal cutting planes^33–36^.

While the large collection of image data in Allen mouse brain connectivity atlas are well suited to visualize axonal trajectories and identify the presence of connections among regions across the entire mouse brain, it is not straightforward to explore principles of spatial organization and 3D distribution patterns in these data, since images can only be viewed or downloaded case by case. While 3D image volumes from multiple experiments in principle could be downloaded and co-displayed in volumetric viewer software, the exploration of labelling patterns is technically cumbersome since data representing the axonal labelling need to be extracted from images, to allow co-visualization in a fashion that facilitates inspection and pattern comparison.

**Fig. 1:**
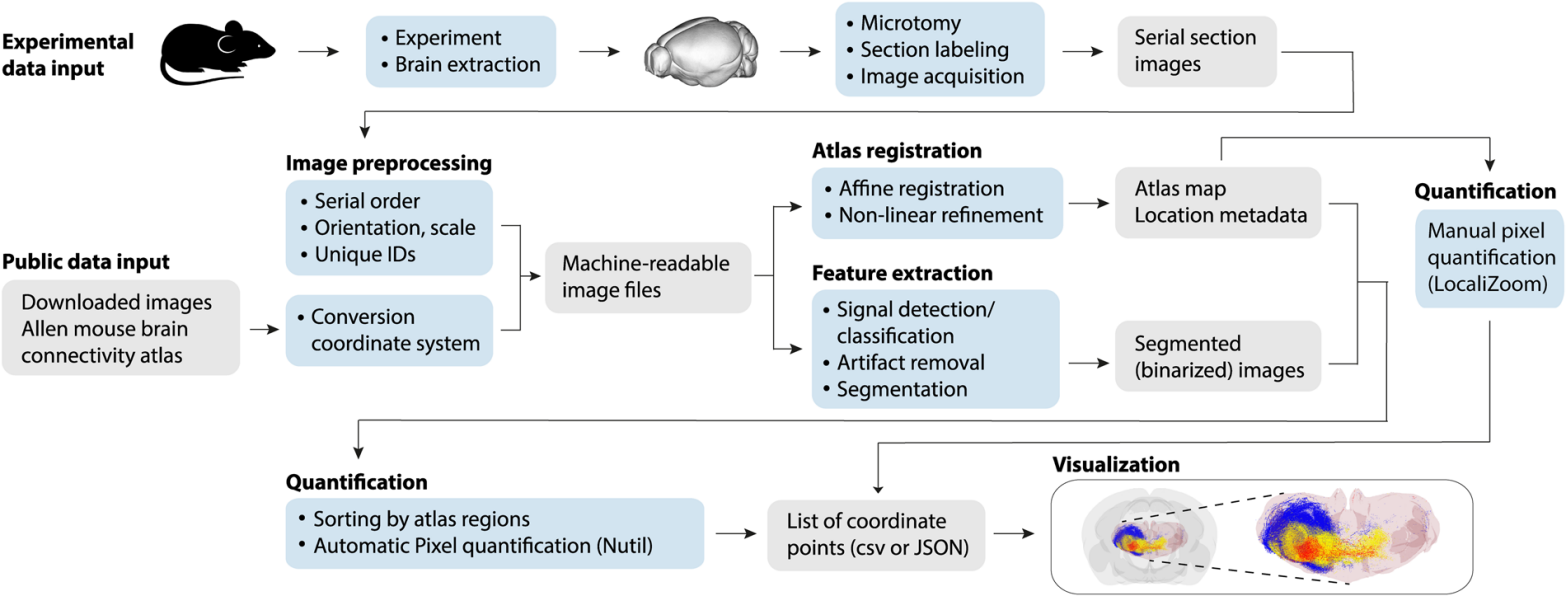
Workflow for deriving atlas-integrated data points. Methodological steps used to extract point coordinate data representing axonal labelling from image data and co-visualizing them in reference atlas space. Inputs to the workflow are either images of serial histological sections generated using tract tracing (top row) or public collections of image data, in our case images downloaded from the Allen mouse brain connectivity atlas. Following image pre-processing steps images are in parallel run through an atlas registration procedure and image extraction procedure. For the images from the Allen Institute, these steps involved validation and adjustment of pre-existing atlas registrations and binarization of pre-existing segmentation images. For automatic extraction of point coordinate data in atlas-defined regions of interest, binary images and corresponding atlas images are processed using the Nutil tool. As an alternative to feature extraction using automated methods, the location of labelling can be recorded by manual annotation using the LocaliZoom tool. The quantification steps produce lists of point coordinates, either as.csv or.json files, representing the extracted feature. The outputs of the workflow are point coordinate data that can be visualized together with atlas structures, e.g. using the MeshView tool, or used for other analytic purposes.

Building on previous efforts to map topographical organization in the cat and rat brain stem nuclei^34,37–44^, we developed a workflow to process Allen mouse brain connectivity atlas 3D image data and convert them into point-cloud data optimized for exploration of spatial distributions of axonal labelling distributions in a common atlas space (Fig. 1). The workflow was tested and validated using tract tracing data from transgenic mice^45^ and images from the Allen mouse brain connectivity atlas^16,19^.

Here we present a collection of point-cloud data representing the 3D spatial distribution of corticostriatal, corticothalamic, corticotectal, and corticopontine projections originating from 46 discrete locations across all major areas in the cerebral cortex of 35 wild-type and 11 transgenic mice. The data points were semi-automatically derived from public data shared by the Allen mouse brain connectivity atlas. The point coordinate data are mapped in version 3 of the Allen mouse brain Common Coordinate Framework (CCFv3) and semi-quantitatively represent anterogradely labelled axonal projections.

We demonstrate how selected combinations of point-cloud data from multiple experiments can be co-displayed and inspected in a 3D viewer, allowing users to interactively inspect and compare spatial distribution patterns using custom viewing angles and arbitrarily cut slices through the point clouds. To exemplify how these spatially integrated point data can be used as references for investigations of molecular and genetic mechanisms underlying topographic organization using genetically engineered animal models, we applied the analytical workflow to map corticopontine projections in transgenic mice lacking postmitotic expression of the area patterning gene *Nr2f1* and littermate controls^45^. Further, for cross-species comparison of corticopontine projections in mice and rats, we utilized a public collection of 3D reconstructed data points representing rat corticopontine projections from several earlier tract-tracing studies^33,35,46^. We describe how the point data can be spatially integrated with user generated experimental data and include Jupyter notebook scripts for generating custom-defined JSON files, based on user-defined experiments and regions of interest that can be visualized in the 3D viewer tool, MeshView.

The public point data collections shown here represent subcortical projections from 172 cerebrocortical locations in mice and rats and are suitable for exploring and comparing topographical organization in mouse brain corticostriatal, corticothalamic, corticotectal, and corticopontine projections, as well as rat brain corticopontine projections.

## Methods

We used a selection of public experimental mouse brain tract-tracing image data^17,18^ downloaded from the Allen Institute mouse brain connectivity atlas (http://connectivity.brain-map.org/), and a collection of 3D point cloud data derived from mouse^47–49^ and rat^50–53^ brain tract-tracing image data, downloaded from the EBRAINS research infrastructure (http://search.kg.ebrains.eu). Our collection includes experimental tract-tracing data from a total of 68 mouse brains (68 tracer injections), and 60 rat brains (104 tracer injections). All data were generated in line with national ethical guidelines as stated in the original publications^16,17,33,35,45,46,54^. The workflow for extracting axonal labelling features from microscope image data and representing these in a common reference atlas space given presented below (Fig. 1), with separate descriptions of the automated and manual methods used to extract 3D point data. Overview of all public experimental data used is provided in Supplementary Table 1, while overview of all new data sets generated and shared in the present study, with digital object identifiers (DOI), is given in Table 1.

**Table 1:** Overview of mouse and rat datasets shared on EBRAINS.

Animals	Number of cases	Number of injection sites	Injection site locations	Data extraction method	Dataset DOI
Mouse wild type	35	35	multiple target regions	Automatic	10.25493/GDYP-B1B
Mouse layer specific Cre lines	11	11	multiple target regions	Automatic	10.25493/HWJY-RDV
Nr2f1 Nex-Cre mice	12	12	corticopontine regions	Manual	10.25493/11HT-S4B
Nr2f1 littermate controls	10	10	corticopontine regions	Manual	
Mouse wild type	11*	11*	corticopontine regions	Manual	
Rat wild type	13	26	S1 whisker barrel cortex	Manual	10.25493/W8MG-X2R
Rat wild type	11	22	Frontal, parietal, temporal and occipital cortical areas	Manual	10.25493/9TMN-64U
Rat wild type	20	40	Sensorimotor cortex	Manual	10.25493/TH1N-V8P
Rat wild type	16	16	Somatosensory cortex	Manual	10.25493/ZSZ9-3NN

* The same 11 animals are included in the collection mouse wild type group of 35 animals, with use of a different method to extract data. For further details, see Supplementary Table 1.

### Semiautomatic extraction of 3D point data representing labelled axons in mouse brain images

#### Selection, downloading, and atlas integration of data

Volumetric image data from anterograde viral tracer experiments conducted in wild-type adult C57BL6/J male mice (n = 35, stock no. 00064, The Jackson Laboratory) were downloaded from the Allen mouse brain connectivity atlas (Supplementary Table 1). In these experiments, focal injections of the pan-neuronal AAV-expressing EGFP viral tracer (rAAV2/1.hSynapsin.EGFP.WPRE.bGH, Penn Vector Core, AV-1-PV1696, Addgene ID 105539) were unilaterally placed in the right cerebral cortex^17,19^, giving rise to anterograde bilateral labelling in subcortical target regions.

Available tracer injection sites were manually inspected using the online Projection High Resolution Image viewer in the Allen Brain Atlas data portal (https://connectivity.brain-map.org/), and the 35 experiments were selected to represent all main cortical areas, including cases in which the tracer injection sites covered all cortical layers, and excluding cases in which the tracer injections involved white matter or several cortical areas. As an additional comparative resource, images from selected experiments conducted in gene-specific promotor-driven Cre mouse lines mice (n = 11) were downloaded and included in the analyses (Supplementary Table 1). Using an application programming interface (API) provided by the Allen Institute and a custom-made python script (https://github.com/Neural-Systems-at-UIO/allen2quicknii), complete series of raw serial two-photon images and derived segmented images for the selected cases were downloaded together with vector coordinates of the registered position of each image in the common coordinate framework version 3, version 2017 (CCFv3_2017)^21^. All images were reoriented to match the orientation used in the 3D MeshView tool (RRID:SCR_017222); coordinates follow the RAS (Right-Anterior-Superior) orientation and are expressed in 25 µm voxels (see also MediaWiki-description of Coordinate Systems on https://www.nitrc.org/projects/quicknii).

The downloaded vector coordinates of the section images provided the spatial registration of images to the CCFv3_2017. The anatomical correspondence of images to reference atlas landmarks was validated using the QuickNII software^43^ (RRID: SCR_016854; version: 2.1; https://quicknii.readthedocs.io). In all sections, minor affine atlas adjustments were made to improve the overall spatial fit of the cerebral cortex, caudoputamen, thalamus, pontine nuclei, and superior colliculus between experimental images and the atlas. These adjustments partially compensated for tissue damages and misalignments, which were most prominent in the cerebral cortex and olfactory bulb.

#### Extraction of point coordinates representing axonal labelling

To reduce image processing time, the raw and segmented images were resized to 10% of their original size image (.PNG file), which gave a good compromise between image resolution, information content and processing speed. Grey contour lines were removed using an ImageJ thresholding function, with additional manual editing, before binarization was performed using a macro function in ImageJ^55^ (RRID:SCR_002285), defining all pixels as signal (i.e. tracer labelling) or background.

Binarized images were then combined with corresponding atlas images exported from the QuickNII using the Nutil Quantifier software^56^ (RRID:SCR_017183; v.0.060; https://nutil.readthedocs.io). Nutil Quantifier was used to extract one point coordinate for each (black) segmented pixel. For the segmentation, objects smaller than 2 pixels were excluded, and an “object splitting” feature was used to sort each pixel to its corresponding atlas region. Since the data were acquired from 2D section images, point coordinates were initially confined to the original orientations of the sections they were recorded from. To facilitate 3D inspection of point populations, the points were randomly distributed within the thickness of the original section planes, using a custom Python script (https://github.com/Neural-Systems-at-UIO/3d-point-clouds/blob/main/code/spread_points.py) that applies a Gaussian transformation function with a standard deviation of 2.5 perpendicular to the plane of sectioning, distributing coordinates within the gaps between the original sections.

### Manual extraction of mouse brain connectivity data

Public tract-tracing image data are well suited as control data in the context of experimental investigations in disease models, but with fluorescence microscopic techniques, image intensities often vary too much for automatic feature extraction to be reliable. Manual recording of axonal labelling as 3D points then provides an alternative for investigations comparing spatial distribution patterns. We here used open-access tract-tracing data from our recent study in transgenic mice of the impact of cortical area patterning genes on topographical organization in the pontine nuclei (n = 12 Nr2f1 Nex-Cre mice; n = 10 Nr2f1 littermate controls), for which tract-tracing image data downloaded from the Allen mouse brain connectivity atlas were used as controls (n = 11 wild type mice)^47^ (Supplementary Table 1). To record location and relative density of labelled axons in images from serial sagittal sections through the pontine nuclei, we used a semiquantitative data recording approach adopted from previous studies^33,54,57,58^, combined with spatial registration to a common reference space^39,43^. Briefly, all section images were first spatially registered to the Allen mouse brain reference atlas (CCFv3) using the QuickNII tool, before using the annotation functionality in the LocaliZoom tool (RRID:SCR_023481; https://localizoom.readthedocs.io) to assign point coordinates at regular intervals over labelled axonal plexuses, reflecting the observed density and location of labelling. In these cases, labelling was recorded only from the right pontine nuclei, ipsilateral to the injection site, where the most predominant labelling was seen. To compensate for the spacing between sections and allow inspection of point distributions perpendicularly to the section angle, the z-coordinate of each point was randomly displaced within the thickness of the gap between sections using a Python script (see, above). For further details, see^47^.

### Rat brain tract tracing data

To demonstrate how the organization of subcortical projections can be compared between mice and rats, we used a collection of 3D-reconstructed point coordinate data representing anterogradely-labelled corticopontine axonal projections originating from frontal, parietal, temporal, or occipital areas of the cerebral cortex^50–53^. These data are based on anterograde tracing experiments performed in adult, male and female Wistar rats, and adult female Sprague Dawley rats, in which one or several anterograde axonal tracers (*Phaseolus vulgaris* leucoagglutinin, Pha; biotinylated dextran amine, BDA; rhodamine conjugated dextran amine; FluoroRuby, Fr; fluorescein conjugated dextran amine, FluoroEmerald, Fe; FluoroGold, FG; Fast Blue, FB, Wheat germ agglutinated horseradish peroxidase, WGA) were applied at various anatomically (and in some cases electrophysiologically) defined discrete locations in the right or (in one series of rat brain experiments) left cerebral cortex (Supplementary Table 1). Anterogradely-labelled axonal clusters and anatomical landmarks observed in the pontine nuclei by light or fluorescence microscopy were bilaterally recorded as points using an image-combining computerized microscope^57^. Data points were 3D reconstructed using the program Micro3D^34^, and linearly registered to a common, standardized coordinate system for the pontine nuclei^39,46,54^ using affine transformations. For methodological details and analysis of topographical patterns of organization, see the original publications^33,35,46,54^. For the first release of the point coordinate data^59–61^, the pontine nuclei coordinate system had been spatially registered to anatomical landmarks observed in the *T*_2_* magnetic resonance image employed in the Waxholm Space atlas of the rat brain (v1.01; 10.25493/DTSG-ZBS)^62^, allowing all x,y,z point coordinates to be translated to Waxholm Space coordinates using a custom Python Script (https://github.com/Neural-Systems-at-UIO/xyz-converter). Waxholm Space rat brain atlas coordinates are based on voxel units with isotropic size of 39 µm, for details about the origin, orientation, and application of the coordinate system, see original documentation and notes on the WHSSDr v1.01 coordinate system provided with the atlas (https://www.nitrc.org/projects/whs-sd-atlas; RRID:SCR_017124). For the present study, we created a script to transform coordinate files from.xyz lists to JSON files (https://github.com/Neural-Systems-at-UIO/3d-point-clouds/blob/main/code/xyz2json.py). To facilitate visualization of data points with the MeshView tool, the xyz points were transformed with a Gaussian function (standard deviation of 2.5) perpendicular to the plane of sectioning to spread out the coordinates within the thickness of the gap between the sections and assigning random colours, using the above-mentioned Python script, outputting JSON files that can be opened in the MeshView tool.

To quantify the spatial difference between two 3D point clouds, we computed the Euclidean distance between point ${A}_{i}=({x}_{{A}_{i}},{y}_{{A}_{i}},{z}_{{A}_{i}})$ in cloud A and every point ${B}_{j}=({x}_{{B}_{j}},{y}_{{B}_{j}},{z}_{{B}_{j}})$ in cloud B. For each *A*_*i*_, the minimum Euclidean distance was selected to identify the nearest neighbouring point in cloud B. The Euclidean distance ${d}_{{A}_{i}-{B}_{j}}$ between point *A*_*i*_ and each point *B*_*j*_ was calculated using the following formula:

$${d}_{{A}_{i}-{B}_{j}}=\mathop{\min }\limits_{j}\sqrt{{({x}_{{B}_{j}}-{x}_{{A}_{i}})}^{2}+{({y}_{{B}_{j}}-{y}_{{A}_{i}})}^{2}+{({z}_{{B}_{j}}-{z}_{{A}_{i}})}^{2}}$$

The distance provides a quantitative measure for “closeness” or “similarity” between points in a multidimensional space, where a value of 0 indicates perfect alignment or overlap between the points. We added a script for the analysis and visualization of the Euclidean distance (https://github.com/Neural-Systems-at-UIO/3d-point-clouds/blob/main/code/calculate_distance.ipynb).

### Mapping of tracer injection sites in reference atlas space

The location of tracer injection sites in the cerebral cortex was defined using stereotaxic, electrophysiological, or histological methods, as specified in the original publications. For each data set, injection site locations were mapped onto common graphical representations, either dorsal view drawings of the mouse and rat brain cortex, body map representations of the primary somatosensory cortex, or semantically for injections placed in electrophysiologically defined whisker representations in rats (see original publications and online data cards for each dataset).

### Data sharing via the EBRAINS infrastructure

Data were organized and curated using the Data and Knowledge Service provided by the EBRAINS research infrastructure (https://www.ebrains.eu/data/share-data). Standardized OpenMinds (RRID:SCR_023173) metadata were assigned to make data discoverable via the EBRAINS Knowledge Graph. The point-coordinate files generated and accompanying metadata, with adjusted CCFv3 registration files (for data from the Allen mouse brain connectivity atlas), were shared under a CC BY 4.0 license.

## Data Records

The point data sets generated and presented in context of this report are shared *via* the EBRAINS research infrastructure (https://search.kg.ebrains.eu) as 3 mouse brain data sets^47–49^ and 4 rat brain data sets^50–53^. Table 1 provides an overview of the shared data files with DataCite DOIs, together comprising data from 172 anterograde neural tracing experiments. For each experiment, we share brain region-specific coordinate files with point-cloud x-, y- and z- coordinates given as plain.txt and.csv format, as well as.json format files tailored for visualization with the MeshView viewer tool (RRID:SCR_017222). For image data downloaded from the Allen Mouse Brain Connectivity Atlas, we also provide text files with vector coordinates representing the improved registration of images to CCFv3 (.xml) for use with the QuickNII tool. The provided point-clouds can be viewed with the MeshView viewer tool.

## Technical Validation

The presented data are based on well-established tract-tracing methods^11,12,63^ and use of semi-quantitative 3D point representations for analysing topographical distribution patterns in axonal tract-tracing experiments^64,65^. The data have previously been used for descriptions of topographic organization in several brain projection systems^21,24,35,57,66,67^, as well as for demonstrating altered neural connections in genetically manipulated mice^45^. While investigations of detailed topographic organization patterns typically have relied on the use of dual tracer injections in the same experiment^68,69^, we previously showed that atlas-based co-registration of data from different single tracing experiments replicates detailed findings of topographical organization previously demonstrated by the use to two axonal tracers^70^. Earlier studies also showed that 3D point representations of labelling yield reproducible descriptions of somatotopic projection patterns in somatosensory related corticopontine pathways in both mice and rats^33,45,46^. Our selection of rat brain corticopontine projection data also includes point clouds from a detailed study of discrete topographic shifts in topographical location within or across rows of whisker representations in the primary somatosensory cortex. Small incremental shifts in the cortical location of tracer injection sites, result in systematic, discrete shifts in the spatial distribution of point clouds representing axonal labelling^54^. This demonstrates that fine-grained spatial distribution patterns can be resolved by accumulation of 3D point data from different experiments.

**Fig. 2:**
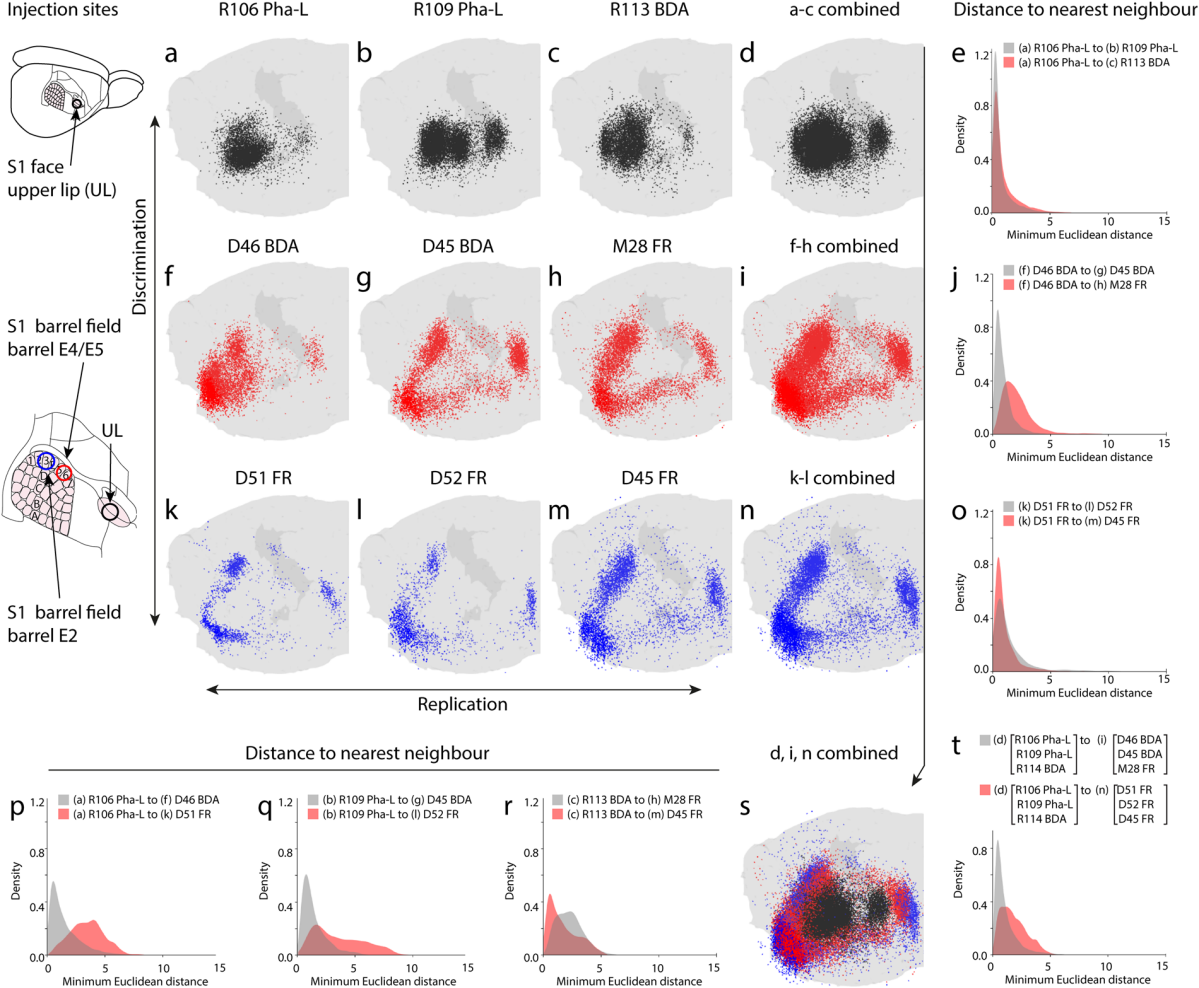
Examples showing reproducibility and variability in the data sets. (**a**–**c,f**–**h,k**–**m**) show 3D visualizations of rat brain data points in the right pontine nuclei, originating from nine tracer experiments in which injections were placed in different S1 body representations, either the S1 upper lip representation (**a**–**c**, black dots), the S1 whisker E4/E5 representation (**f**–**h**, red dots) or the S1 whisker E2 representation (**k–m**, blue dots). Injection site locations in the rat cerebral cortex are illustrated in the left column. (**d,i,n**) show data points combined from three similar experiments (organized in rows), revealing that the cases together occupy a slightly more expansive space in the pontine nuclei. This reflects biological and methodological variability in the data, and the degree with which findings can be replicated. Compared across columns, the data show that moderate displacement of cortical injection sites, from S1 upper lip to the S1 whisker E4/5 (~2,5 mm distance) or S1 whisker E2 (~3,5 mm distance) representation, result in consistent shifts in the distribution of the point clouds. This reflects the granularity of the data, and the degree with which the data allow discrimination of topographical patterns. This is visible in panel **s**, showing the colour coded data from all nine cases, concentrically distributed in an inside-out fashion. Graph plots in the right column (**f,j,o**) and bottom row (**p**–**r**) show measurements of the minimum Euclidean distance between a point in one cloud and the nearest point in another cloud. Distance units are Waxholm Space voxel coordinates, 1 voxel = 39 µm isotropic. Plots in the right column shows measurements from cases in the left column (**a,f,k**) to the other two data sets in the same row, and plots in the bottom row showing measurements from cases in the top row (**a,b,c**) to the other two data sets in the same column. The plot in panel t shows the measurements from the accumulated data points in d to the accumulated data shown in i and n. The nearest neighbour measurements show that the average Euclidean distance is low when comparing cases with similar injection sites, and slightly higher when comparing cases with dissimilar injection sites. Abbreviations: BDA, biotinylated dextran amine; FR, FluoroRuby; Pha-L, *Phaseolus vulgaris* leucoagglutinin; UL, upper lip. Scale bars, 1 mm.

The results of tract-tracing experiments are influenced by several experimental variables (see, e.g. reviews by Lanciego and Wouterlood^11,12^), including the position and extent of the effective injection site, the efficacy of tracer uptake and transport, and for the data presented here, also the methods used for data recording and spatial co-registration. The location of the tracer injection sites determines which parts of the underlying neural network are visualized and serves as the primary experimental parameter of interest. While the variability in the data is difficult to quantify, visualization of pontine nuclei point clouds from experiments with similar injection sites, e.g. located in the same S1 body representation in the rat cerebral cortex, are highly consistent^33^ (Fig. 2, rows a-d, f-I, k-n). Methodological sensitivity is highlighted by the observation that moderate shifts in cortical injection site location (~2.5–3.5 mm, see, e.g. S1 map in Leergaard *et al*.^46^) within a cortical region or subregion, result in topographically predicable shifts of point distributions in the pontine nuclei^54^ (Fig. 2, columns a-f-k, b-g-l, c-h-m, d-i-n).

A quantitative measure for the similarity of point clouds in a multidimensional space can be provided by the minimum Euclidean distance between a point in one cloud and the nearest point in another cloud^71,72^ (Fig. 2e,j,o,p-r,t). The Euclidean distances between points clouds in two data sets can be used as an approximation of similarity. It assumes that there is a one-to-one correspondence between points in both clouds, which is not the case for the current examples due to experimental and methodological differences. The Euclidean distance is also highly sensitive to noise and dispersed point clouds may cause disproportionally short or large distances that skew the overall results. Nevertheless, to provide an approximation of replicability and variability in our data, we performed an example analysis using our rat corticopontine data. When comparing point clouds arising from corresponding tracer injections in the same S1 body representations, we find short average minimum Euclidean distances, illustrated by narrow density curves close to zero (Fig. 2e,j,o). In contrast, the average Euclidean distance between point populations representing corticopontine projections arising from injections in different S1 body representations (upper lip and different whisker barrels) is larger and more dispersed, illustrated by wider density plots with a longer tail (Fig. 2p–r,t). Although these measurements should be interpreted with caution, the plots in Fig. 2 show that experiments with similar injection sites have a higher degree of similarity (i.e. shorter Euclidean distances) compared to those with injection sites in separate cortical locations.

**Fig. 3:**
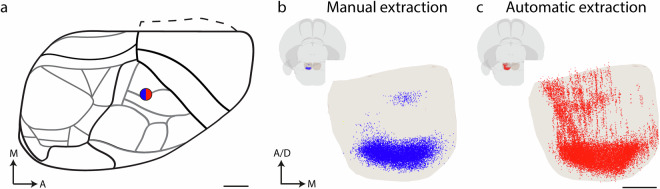
Comparison of manual and automated feature extraction methods. 3D visualization of data points representing corticopontine projections in an experiment downloaded from the Allen Mouse Brain Connectivity Atlas, in which a tracer injection was placed in the primary somatosensory cortex (case #112229814). (**a**) shows a dorsal view diagram of the right mouse brain hemisphere, with the injection site location indicated by a blue/red circle. (**b**) and (**c**) show the pontine nuclei from the Allen mouse brain atlas (CCFv3) rendered as a transparent surface in view from anteroventral, with manually (**b**, blue points) and automatically (**c**, red points) extracted point representing corticopontine projections observed in this experiment. The visualized point clouds feature an overall comparable topographic distribution pattern, with a dense, horizontally oriented cluster caudally in the pontine nuclei, and a smaller, rostrally located cluster, but the comparison shows that automatic feature extraction method yields more abundant and widely distributed data points in some regions, including more labelled axons and thus emphasizing regions with relatively low labelling densities. Abbreviations: A, anterior; D, dorsal; M, medial. Scale bars: (A) 1 mm, (B) and (C): 200 µm.

Feature extraction methods differ in sensitivity. Automated methods relying on computationally defined thresholding and filtering may give variable results depending on the signal intensity and background present in experimental images. Manual methods for recording locations of visually observed labelling can compensate for intensity variation and are less prone to include background noise. This results in systematic differences in the density of labelling recorded, particularly in regions with low density or scarce labelling. These differences are exemplified in Fig. 3, comparing the corticopontine labelling recorded using manual annotation (Fig. 3b) with automated computational signal extraction (Fig. 3c) in the same experiment. While the automated method reflects a more extensive distribution of labelling, the manual method captures the distribution of the most prominent labelling, which can be advantageous when investigating spatial distribution patterns. The comparison clearly shows that the variations in the sensitivity of data acquisition methods should be considered when comparing and interpreting results.

**Fig. 4:**
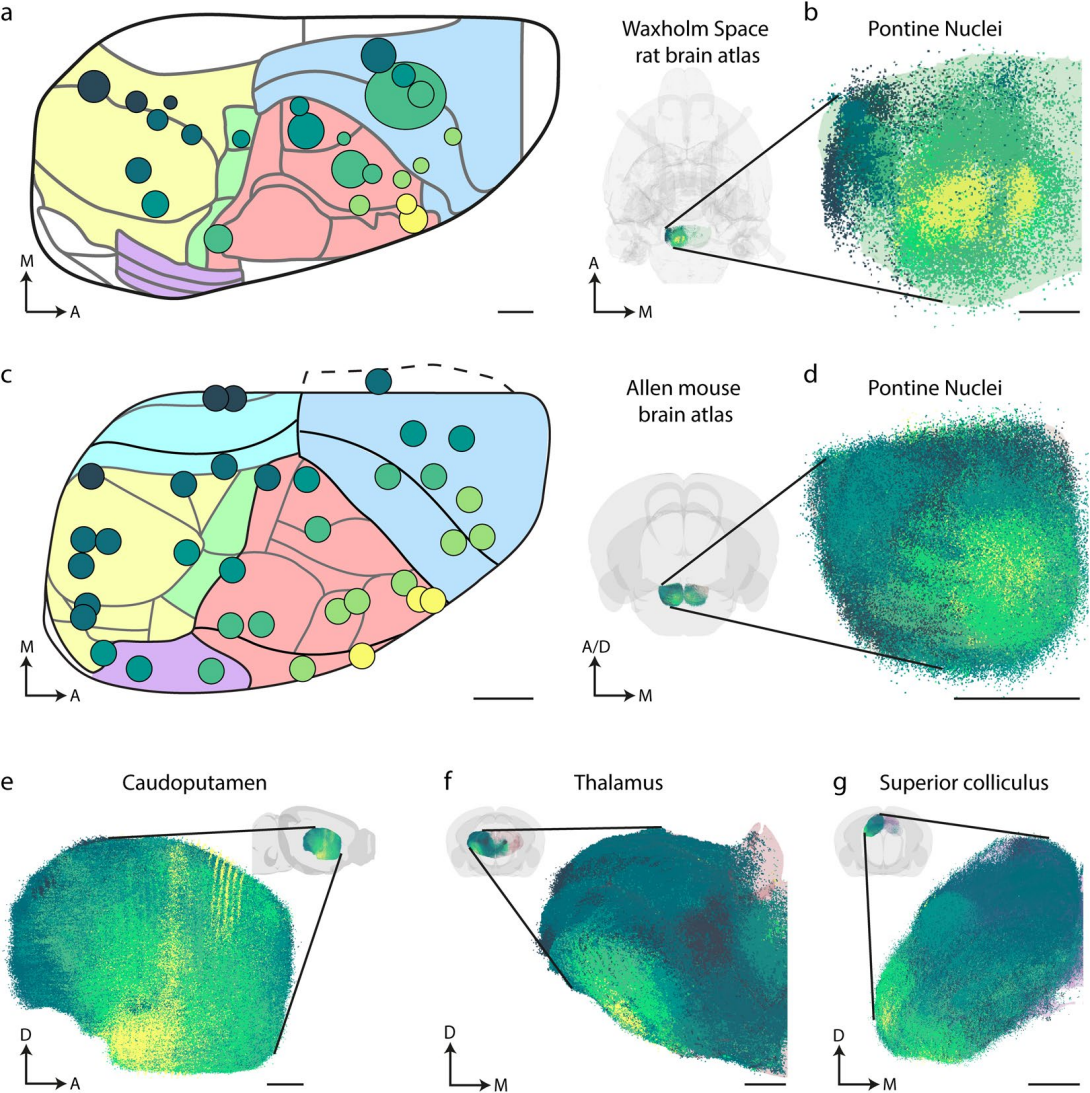
Colour coded visualization of injection sites and data point populations in mouse and rat brains reveals graded topographic distribution patterns. Visualizations of colour coded 3D point data representing corticopontine projections from 24 cortical injection sites in the rat brain (**b**), and corticopontine, corticostriatal, corticothalamic, and corticotectal projections from 35 cortical injection sites in the mouse brain (**d–g**). Diagrams show the right hemispheres of the rat (**a**) and mouse (**c**) brain in dorsal view, indicating the location of injection sites (coloured circles) in relation to different brain regions. Regions in the Waxholm Space rat brain atlas v.4 are colour coded as following: blue (primary and secondary motor cortex with frontal association area 3); pink (primary somatosensory cortex); green (posterior parietal cortex); yellow (visual/occipital cortex); purple (auditory cortex). The regions in the Allen mouse brain atlas (CCFv3_2017) are colour coded as following: blue (somatomotor areas); turquoise (retrosplenial area); red (somatosensory areas); green (posterior parietal association areas); purple (auditory areas and temporal association areas), yellow (visual areas). (**b**) shows the cortical projections to the pontine nuclei in the Waxholm Space rat brain atlas v.4, while (**d–g**) show cortical projections to different subcortical regions in Allen mouse brain atlas CCFv3 (including the pontine nuclei, caudoputamen, thalamus and superior colliculus) rendered as transparent surfaces. The inset transparent surface models of the atlases indicate the orientations in which data points are displayed. The data are colour coded according to the cortical location of injection sites, following a gradient from anterolateral (yellow) to progressively more frontal, medial, and occipital locations (increasingly darker shades of green). The visualized point clouds show that the subcortical projections are topographically organized, with concentric patterns in the pontine nuclei and thalamus, and more linear, fan-like distributions in the caudoputamen and superior colliculus. Abbreviations: M, medial; A, anterior; D, dorsal. Scale bars: A and C, 1 mm; B and D-G, 500 µm.

The present data collection further shows that corticopontine connections are similarly organized in mice and rats^45,73^ (Fig. 4a–d), indicating that the biological cross-species differences are relatively small, and that findings in mice subcortical networks of mice likely can be extrapolated to rats and vice versa.

## Usage Notes

### Visualizing and comparing point data

The presented collections of derived data points are suitable for exploring patterns of spatial distribution in mouse brain corticostriatal, corticothalamic, corticotectal, and corticopontine projections, and for visualizing and comparing the spatial organization of corticopontine projections in mice and rats. The point data can be downloaded as XYZ lists that can be opened in any XYZ file viewer software but are also provided as JSON files configured for 3D visualization with the MeshView tool, which is available with embedded structure annotation meshes from the CCFv3 mouse brain atlas or Waxholm Space rat brain atlas v4 (see Code availability). Selected combinations of pseudo-coloured points can be visualized in MeshView together with atlas meshes, allowing interactive inspection of point clouds and digital slicing through the point clouds in used defined angles of view (See MeshView user manual for more details; https://meshview-for-brain-atlases.readthedocs.io/en/latest/).

**Fig. 5:**
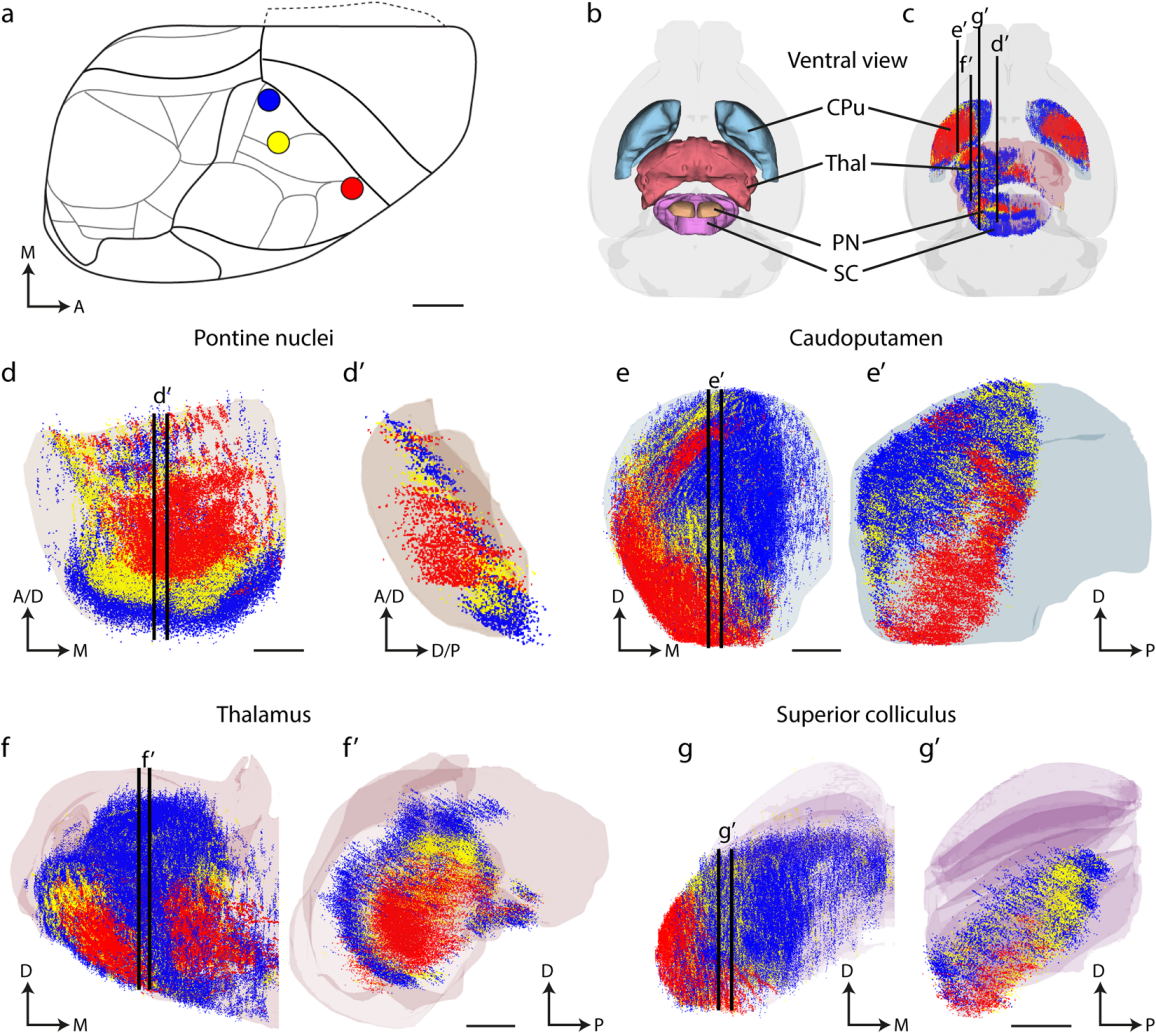
Comparison of topograpical organization in corticopontine, corticostriatal, corticothalamic, and corticotectal projections in the mouse brain. Visualizations of colour coded 3D point data representing subcortical projections from 3 selected tract-tracing experiments in the mouse somatosensory cortex. The diagram in (**a**) shows the right mouse brain hemisphere in dorsal view, with coloured circles indicating the location of tracer injections in the mouth (red, case #114290938), upper limb (yellow, case #112229814), and lower limb (blue, case #114292355) representations in the primary somatosensory cortex. The Allen mouse brain atlas (CCFv3) is shown in view from ventral in (**b**) and (**c**), with the four main subcortical target regions rendered as solid (**b**) or transparent surfaces (**c**). Subcortical projections from the three experiments are co-visualized as colour-coded points in the pontine nuclei (**d**, viewed *én face*, obliquely from rostroventral), caudoputamen (**e**, rostral view), thalamus (**f**, rostral view), and superior colliculus (**g**, rostral view). For each region, a 50 µm thick sagittal slice is cut through the point clouds, at levels indicated with lines in (**c**). Viewed from medial, the slices reveal different concentric or graded patterns of spatial distribution (d’-g’). Abbreviations: M, medial; A, anterior; D, dorsal; P, posterior; CPu, caudoputamen; Thal, thalamus; PN, pontine nuclei; SC, superior colliculus. Scale bars: A, 1 mm; B and C, 2 mm; D, D’, 200 µm; E - G’, 500 µm.

**Fig. 6:**
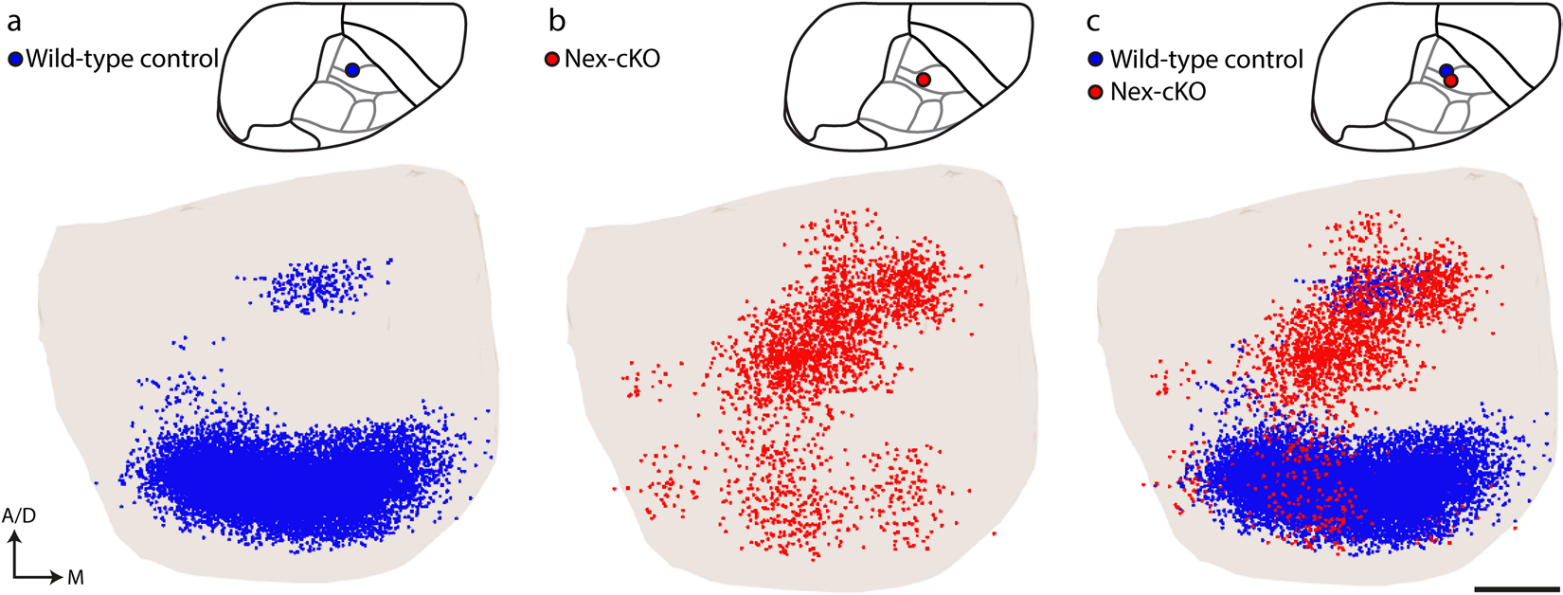
Altered topographical distribution of corticopontine projections in transgenic mice lacking the area patterning gene Nr2f1. The example illustrates how co-visualization of atlas-integrated data points representing corticopontine projections can be used to demonstrate topographical shifts in the distribution of axons in mice lacking the area patterning gene *Nr2f1*, which is known to contribute to the establishment of topographically organized neural networks. 3D visualization of data points representing corticopontine projections from closely corresponding locations in the primary somatosensory cortex (S1) in a wild-type control mice (**a**, case #112229814, blue points) and a Nex-*cKO* mutant mouse lacking Nr2f1 expression (**b**, case #19423_7, red points). The data points are shown within a transparent surface rendering of the pontine nuclei in view from ventral. Co-visualization of the data points (**c**) show that corticopontine projections from the S1 upper limb representation are clearly shifted to a more rostral location in the pontine nuclei, compared to the wild-type control. For a detailed analysis of the altered subcortical projections in mice lacking Nr2f1, see original paper by Tocco *et al*.^45^. Abbreviations: D, dorsal; M, medial. Scale bars: 200 µm.

Data can be selected based on injection site locations, specified semantically or graphically in the online data cards, and opened in a viewer tool. By selecting cases and assigning colours to them, users can address specific questions e.g., based on functional or geometric patterns related to the injection site locations. The examples presented below are visualized using the MeshView tool. MeshView provides the users two options for loading point clouds: 1) load an XYZ coordinate point lists with custom RGB colour codes via “cloud from coordinates”, or 2) load.JSON files with pre-assigned RBG colours via “choose file”. For the exploration of subcortical projections from different cortical regions in wild-type mice and rats, we also developed Jupyter notebooks (https://github.com/Neural-Systems-at-UIO/3d-point-clouds/tree/main/code) allowing users to reproduce the visualizations shown in Figs. 2–6, or modify these to create their own custom JSON files to load specific selections of data from multiple experiments and visualize data points in one or all target regions of interest (see Code availability). Finally, by utilizing the data annotation functionality in the LocaliZoom tool (https://localizoom.readthedocs.io/en/latest/), it is possible to create custom new data points from other experimental data that have been registered to the CCF3 mouse brain atlas or the Waxholm Space rat brain atlas and compare these with the tract tracing data presented here. Interactive 3D inspection of pseudo-coloured data points is important for exploring spatial distribution patterns. When viewing many multi-coloured data points simultaneously, it is helpful to digitally slice the point clouds in user-defined sections that allow finer dissection of distribution patterns (Fig. 5, compare d-g and d’-g’). MeshView also allows users to adjust the point size and toggle data sets, which facilitates the interrogation of different patterns.

Below, we exemplify how the collection of atlas integrated data points can be utilized for comparing spatial distribution patterns and exploring topographical organization across different subcortical projection systems in mice, to compare corticopontine projections in mice and rats, or to use wild-type projection data as benchmarks for investigating possible alteration of neural connections in transgenic mice or other experimental data.

### Graded topographical organization in mouse and rat subcortical projections

The starting point for exploring spatial patterns in the data is to select and colour code data based on the location of tracer injection sites in the cerebral cortex, either graphically using the injection site maps provided in the data cards, or semantically using the tabular specifications of injection site areas (Supplementary Table 1).

In our first example, we used the mouse brain data to demonstrate the overall inside-out concentric topographical organization of corticopontine projections described in rat^33,35,54,57,73^.This organization is based on the ‘chrono-architectonic’ hypothesis stating that early generated neurons in the anterolateral cerebral cortex innervate early generated pontocerebellar neurons in the central core of the pontine nuclei, while later generated cortical neurons, located closer to the frontal, medial, and occipital parts of the cortex, innervate later generated pontocerebellar neurons located more peripherally in the pontine nuclei^35,57,74^. We thus colour coded a large selection of rat and mouse brain injection sites along the neurogenetic gradient^75^ and graded gene expression patterns^76^ described in the mouse cerebral cortex. We assigned a yellow to dark green colour gradient to cases according to their distance from the anterolateral cerebral cortex towards frontal, medial, and occipital locations (Fig. 4a,c). Data points in the pontine nuclei showed projections from the anterolateral cortex (yellow points) distributed centrally, while projections from cortical locations closer to the midline (gradually darker shades of green), are distributed more peripherally in the pontine nuclei (Fig. 4b,d). The concentric gradient patterns are consistently observed in both species. Since the mouse brain data sets include data from other brain regions, topographical organization can also be compared across regions by applying the same colour coding to visualize data points in the caudate-putamen complex, thalamus, and superior colliculus. The corticopontine and corticothalamic projections overall display concentric, inside-out arrangements, while corticostriatal, and corticotectal projections appear to be clustered in graded, fan-like ventral to dorsal arrangements (Fig. 4e,g). These observations highlight that the present point data collection can be used to investigate more detailed features of topographical organization in these subcortical circuits.

To further showcase how these patterns can be explored in more detail, we selected three mouse brain injection sites placed at increasing distances from the anterolateral cortex, from the perioral face representation in S1 to injections in the forelimb and hindlimb representations of the cerebral cortex (Fig. 5a). For this analysis we also ‘dissected’ the data by applying sagittally oriented virtual slices through the core of the data clouds to more clearly see spatial distribution patterns in the coloured point clouds. The 3D visualizations reveal a distinct concentric arrangement from central (red points) to more peripheral locations (yellow and blue points), with axons arising from the S1 upper lip region (red points) projecting slightly lateral to the core of the pontine nuclei, while projections arising from the forelimb (yellow points), and hindlimb (blue points) representations are distributed in progressively more peripheral and predominantly caudal parts of the pontine nuclei (Fig. 5d-d’).

### Evaluating spatial distribution patterns in a transgenic disease model

The shared data representing the spatial organization of neural connection in wild type animals from the Allen Brain Connectivity Atlas are suitable as benchmark references for exploring the effects of genetic perturbations. We compared data extracted with a semi-quantitative manual approach from Nr2f1 mutant mice, with data from corresponding experiments in littermate controls or wild type mice from the Allen mouse brain connectivity atlas that were extracted using the same method. 3D visualization of point data representing corticopontine projections from tracer injections placed in the same cortical location, such as the forelimb representation of S1, reveal that projections in mutant animals are shifted to more rostral locations compared to littermate controls (Fig. 6). More examples of such analyses are provided by Tocco *et al*.^45^.

### Adding information from other experimental data

Since all 3D point data are integrated into standard reference atlas spaces, they are interoperable with spatial information from other types of image data that have been registered to the same reference atlas and can be co-visualized in a 3-D viewer. Data should preferably be extracted using the same method to reduce methodological variability. For example, the LocaliZoom tool as described above, provides a low-threshold and straightforward procedure to record data points from new experimental data or public data registered to the same atlas. Examples of such analyses are provided by Leergaard and Bjaalie^73^.

## Supplementary information

Supplementary Table 1

## Data Availability

All data are shared via the EBRAINS research infrastructure as specified in the Data records paragraph above. Image J is available from https://fiji.sc/. The software tools QuickNII (RRID:SCR_016854), VisuAlign (RRID:SCR_017978), LocaliZoom (RRID:SCR_023481), Nutil (RRID: SCR_017183), and Meshview (RRID:SCR_017222) are open source and freely accessible at the EBRAINS infrastructure (https://www.ebrains.eu/tools?filter=brain-atlas), together with user manuals or links to webservices. The following Python scripts used in the present work are available via Github: - Custom script for downloading images from the Allen Brain Institute: https://github.com/Neural-Systems-at-UIO/allen2quicknii - Script for spreading point coordinate points in JSON files randomly across a section thickness: https://github.com/Neural-Systems-at-UIO/3d-point-clouds/blob/main/code/spread_points.py - Script for converting x,y,z point lists to JSON files compatible with MeshView: https://github.com/Neural-Systems-at-UIO/3d-point-clouds/blob/main/code/xyz2json.py - Script for the comparing the similarity between clouds by calculating the Euclidean distance (Fig. 2): https://github.com/Neural-Systems-at-UIO/3d-point-clouds/blob/main/code/calculate_distance.ipynb - Script for the comparison of feature extraction methods (Fig. 3): https://github.com/Neural-Systems-at-UIO/3d-point-clouds/blob/main/code/compare_extraction_method.ipynb - Script for colour coded visualization of injection sites in the wildtype mouse (Fig. 4): https://github.com/Neural-Systems-at-UIO/3d-point-clouds/blob/main/code/create_gradientmap_WT.ipynb - Script for colour coded visualization of injection sites in the rat (Fig. 4): https://github.com/Neural-Systems-at-UIO/3d-point-clouds/blob/main/code/create_gradientmap_rat.ipynb - Script for comparing points representing projections to different subcortical regions in the same animals (Fig. 5): https://github.com/Neural-Systems-at-UIO/3d-point-clouds/blob/main/code/compare_regions.ipynb - Script for comparing mouse brain connectivity points in different mouse genotype (Fig. 6): https://github.com/Neural-Systems-at-UIO/3d-point-clouds/blob/main/code/compare_genotype.ipynb
