# Supplementary material for: Spatially integrated cortico-subcortical tracing data for analyses of rodent brain topographical organization
Source: Sci Data. 2024 Nov 12;11:1214. doi: 10.1038/s41597-024-04060-y (PMC11557934; doi:10.1038/s41597-024-04060-y)
Supplement: Supplementary file 1 — Supplementary Table 1 [file 41597_2024_4060_MOESM1_ESM.pdf]

This overview table contains the following metadata for each dataset:

**expNumber**	Experiment number
**expName**	Experiment name
**genotype/strain**	Genotype and strain name
**sex**	Sex of the animal
**age_injectio**	Age at tracer injection timepoint
**age_euthanisia**	Age at euthanasia
**injectionArea**	Abreviated name of the brain region where the tracer was injected
**regionFull**	Full name of the brain region where the tracer was injected
**injection_hemisphere**	Hemisphere of tracer injection
**colorName**	Color name of the injection site and point clouds representing anterogradely labelled axons
**RGB**	RGB color code
**gradient**	Color coding of the injection site according to their distance from the anterolateral cerebral cortex towards frontal, medial, and occipital locations (fig3 A, C)<br>1: (RGB 250 250 110); 2: (RGB 156 223 124); 3: (RGB 74 189 140); 4: (RGB 0 150 142); 5: (RGB 16 110 124); 6: (RGB 42 72 88)

RGB: 250 250 110	RGB: 156 223 124	RGB: 74 189 140	RGB: 0 150 142	RGB: 16 110 124	RGB: 42 72 88
HEX: #fafa6e	HEX: #9cdf7c	HEX: #4abd8c	HEX: #00968e	HEX: #106e7c	HEX: #2a4858

Mouse wild t WT https://doi.org/10.25493

expNumber	expName	genotype	strain	sex	age_injection	age_euthanisia	injectionArea	regionFull	injection_hemisphere	colorName	RGB	gradient
1	100140949	C57BL/6J	NA	male	P56	10 weeks	RSPv	Retrosplenial area, ventral part	right	darkolivegreen	[85 107 47]	6
2	100141219	C57BL/6J	NA	male	P56	10 weeks	VISp	Primary visual area	right	mediumslateblue	[123 104 238]	5
3	100141473	C57BL/6J	NA	male	P56	10 weeks	SSpbfd	Primary somatosensory area, barrel field	right	lightslategray	[119 136 153]	3
4	100141495	C57BL/6J	NA	male	P56	10 weeks	SSptr	Primary somatosensory area, trunk	right	forestgreen	[34 139 34]	5
5	100141599	C57BL/6J	NA	male	P56	11 weeks	VISam	Anteromedial visual area	right	orange	[255 165 0]	5
6	100141780	C57BL/6J	NA	male	P56	12 weeks	MOp	Primary motor area	right	fuchsia	[255 0 255]	2
7	100147853	C57BL/6J	NA	male	P56	11 weeks	VISp	Primary visual area	right	lightblue	[173 216 230]	5
8	100148142	C57BL/6J	NA	male	P56	11 weeks	RSPv	Retrosplenial area, ventral part	right	maroon3	[176 48 96]	6
9	100149109	C57BL/6J	NA	male	P56	11 weeks	AUDp	Primary auditory area	right	yellowgreen	[154 205 50]	3
10	100149969	C57BL/6J	NA	male	P56	11 weeks	SSpm	Primary somatosensory area, mouth	right	deeppink	[255 20 147]	1
11	112229103	C57BL/6J	NA	male	P56	11 weeks	RSPagl	Retrosplenial area, lateral agranular part	right	tan	[210 180 140]	5
12	112229814	C57BL/6J	NA	male	P56	11 weeks	SSpul	Primary somatosensory area, upper limb	right	darkmagenta	[139 0 139]	3
13	112373124	C57BL/6J	NA	male	P56	11 weeks	SSpn	Primary somatosensory area, nose	right	turquoise	[64 224 208]	2
14	112952510	C57BL/6J	NA	male	P56	11 weeks	MOs	Secondary motor area	right	khaki	[240 230 140]	3
15	113887162	C57BL/6J	NA	male	P56	11 weeks	VISp	Primary visual area	right	peru	[205 133 63]	4
16	114250546	C57BL/6J	NA	male	P56	11 weeks	VISl	Lateral visual area	right	darkcyan	[0 139 139]	5
17	114290938	C57BL/6J	NA	male	P56	11 weeks	SSpm	Primary somatosensory area, mouth	right	navy	[0 0 128]	2
18	114292355	C57BL/6J	NA	male	P56	11 weeks	SSpll	Primary somatosensory area, lower limb	right	lightgreen	[144 238 144]	4
19	117298988	C57BL/6J	NA	male	P56	11 weeks	SSs	Supplemental somatosensory area	right	yellow	[255 255 0]	2
20	126907302	C57BL/6J	NA	male	P56	11 weeks	SSpbfd	Primary somatosensory area, barrel field	right	lightcoral	[240 128 128]	4
21	126908007	C57BL/6J	NA	male	P56	11 weeks	SSpn	Primary somatosensory area, nose	right	plum	[221 160 221]	2
22	127084296	C57BL/6J	NA	male	P56	11 weeks	MOp	Primary motor area	right	cornflower	[100 149 237]	3
23	127866392	C57BL/6J	NA	male	P56	11 weeks	SSpbfd	Primary somatosensory area, barrel field	right	deepskyblue	[0 191 255]	3
24	139426984	C57BL/6J	NA	male	P56	11 weeks	ACAd-ACAv	Anterior cinglate area, dorsal part	right	olive	[128 128 0]	5
25	141602484	C57BL/6J	NA	male	P56	11 weeks	MOs	Secondary motor area	right	limegreen	[50 205 50]	3
26	141603190	C57BL/6J	NA	male	P56	11 weeks	MOs	Secondary motor area	right	pink	[255 192 203]	4
27	146858006	C57BL/6J	NA	male	P56	11 weeks	AUDp	Primary auditory area	right	tomato	[255 99 71]	4
28	157062358	C57BL/6J	NA	male	P56	11 weeks	VISpor	Postrhinal area	right	darkseagreen	[143 188 143]	4
29	157654817	C57BL/6J	NA	male	P56	11 weeks	SSpm	Primary somatosensory area, mouth	right	darkred	[139 0 0]	1
30	174360333	C57BL/6J	NA	male	P56	11 weeks	SSs	Supplemental somatosensory area	right	darkslateblue	[72 61 139]	1
31	180296424	C57BL/6J	NA	male	P56	11 weeks	VISp	Primary visual area	right	blueviolet	[138 43 226]	5
32	272697944	C57BL/6J	NA	male	P56	11 weeks	MOp	Primary motor area	right	blue	[0 0 255]	2
33	272782668	C57BL/6J	NA	male	P56	11 weeks	VISp	Primary visual area	right	violet	[238 130 238]	6
34	307320960	C57BL/6J	NA	male	P56	79 days	VISp	Primary visual area	right	chartreuse	[127 255 0]	5
35	585025284	C57BL/6J	NA	male	P56	79 days	MOs	Secondary motor area	right	red	[255 0 0]	2

Mouse layer Transgenic https://doi.org/10.25493/HWJY-RDV

expNumber	expName	genotype	strain	sex	age_inject	age_euthani	injectionArea	regionFull	injection_	colorName	RGB	gradient
1	159888336	Scnn1a-Tg3-Cre	B6.C3H	female	P56	11 weeks	SSpbfd	Primary somatosensory area, barrel field	right	saddlebrown	[139 69 19]	2
2	166269090	Scnn1a-Tg3-Cre	B6.C3H	female	P56	11 weeks	RSPv	Retrosplenial area, ventral part	right	green	[0 128 0]	6

3	182089608	Scnn1a-Tg3-Cre	B6.C3H	female	P56	11 weeks	VISp	Primary visual area	right	steelblue	[70 130 180]	5
4	287599685	Trib2-F2A-CreERT2	B6.129	female	P56	11 weeks	ACAd	Anterior cingulate area, dorsal part	right	indigo	[75 0 130]	5
5	297652799	Rbp4-Cre_KL100	C57BL/6J	female	P56	11 weeks	SSpll	Primary somatosensory area, lower limb	right	red	[255 0 0]	4
6	297946935	Sim1-Cre_KJ18		female	P56	11 weeks	MOs	Secondary motor area	right	lime	[0 255 0]	2
7	297948420	Sim1-Cre_KJ18		female	P56	75 days	SSpm	Primary somatosensory area, mouth	right	aqua	[0 255 255]	2
8	297951732	Sim1-Cre_KJ18		female	P56	75 days	SSpul	Primary somatosensory area, upper limb	right	blue	[0 0 255]	2
9	303615412	Tlx3-Cre_PL56		female	P56	11 weeks	SSpul	Primary somatosensory area, upper limb	right	laserlemon	[255 255 84]	2
10	591612976	Tlx3-Cre_PL56		female	P56	82 days	MOp	Primary motor area	right	hotpink	[255 105 180]	3
11	606785720	Rbp4-Cre_KL100		female	P56	12 weeks	MOs	Secondary motor area	right	bisque	[255 228 196]	3

Mouse wild t Nex-cKO https://doi.org/10.25493/11HT-S4B

Nr2fl littermate controls

Nr2flNex-Cre mice

**expNumber**	**expName**	**genotype**	**strain**	**sex**	**age_inject**	**age_euthani**	**injectionArea**	**regionFull**	**injection**	**colorName**	**RGB**	**gradient**
1	112952510	C57BL/6J	NA	male	P56	11 weeks	MOs	Secondary motor area	right	black	[0 0 0]	4
2	141603190	C57BL/6J	NA	male	P56	11 weeks	MOs	Secondary motor area	right	black	[0 0 0]	4
3	141602484	C57BL/6J	NA	male	P56	11 weeks	MOs	Secondary motor area	right	black	[0 0 0]	3
4	127084296	C57BL/6J	NA	male	P56	11 weeks	MOp	Primary motor area	right	black	[0 0 0]	3
5	114292355	C57BL/6J	NA	male	P56	11 weeks	SSpll	Primary somatosensory area, lower limb	right	black	[0 0 0]	4
6	585025284	C57BL/6J	NA	male	P56	79 days	MOs	Secondary motor area	right	black	[0 0 0]	2
7	100141780	C57BL/6J	NA	male	P56	12 weeks	MOp	Primary motor area	right	black	[0 0 0]	2
8	112229814	C57BL/6J	NA	male	P56	11 weeks	SSpul	Primary somatosensory area, upper limb	right	black	[0 0 0]	3
9	114290938	C57BL/6J	NA	male	P56	11 weeks	SSpm	Primary somatosensory area, mouth	right	black	[0 0 0]	2
10	126908007	C57BL/6J	NA	male	P56	11 weeks	SSpn	Primary somatosensory area, nose	right	black	[0 0 0]	2
11	127866392	C57BL/6J	NA	male	P56	11 weeks	SSpbfd	Primary somatosensory area, barrel field	right	black	[0 0 0]	3
12	11643_13	Nex-cKO-Neg	NA	NA	P4-P5	P21	MOs	Secondary motor area	right	gray	[102 102 102]	4
13	18035_7	Nex-cKO-Neg	NA	NA	P4-P5	P21	MOs	Secondary motor area	right	gray	[102 102 102]	3
14	18035_2	Nex-cKO-Neg	NA	NA	P4-P5	P21	MOs	Secondary motor area	right	gray	[102 102 102]	3
15	19423_3	Nex-cKO-Neg	NA	NA	P4-P5	P21	MOp-MOs	Primary and secondary motor area	right	gray	[102 102 102]	4
16	11796_2	Nex-cKO-Neg	NA	NA	P4-P5	P21	MOs	Secondary motor area	right	gray	[102 102 102]	3
17	18035_1	Nex-cKO-Neg	NA	NA	P4-P5	P21	MOs	Secondary motor area	right	gray	[102 102 102]	3
18	11796_9	Nex-cKO-Neg	NA	NA	P4-P5	P21	MOp-MOs	Primary and secondary motor area	right	gray	[102 102 102]	3
19	11796_8	Nex-cKO-Neg	NA	NA	P4-P5	P21	MOp-MOs	Primary and secondary motor area	right	gray	[102 102 102]	3
20	19423_2	Nex-cKO-Neg	NA	NA	P4-P5	P21	MOp-MOs	Primary and secondary motor area	right	gray	[102 102 102]	3
21	11431_6	Nex-cKO-Neg	NA	NA	P4-P5	P21	SSpu-SSpbfd	Somatosensory Cortex	right	gray	[102 102 102]	3
22	11643_16	Nex-cKO-Pos	NA	NA	P4-P5	P21	MOs	Secondary motor area	right	red	[255 0 0]	4
23	18035_4	Nex-cKO-Pos	NA	NA	P4-P5	P21	MOs	Secondary motor area	right	red	[255 0 0]	4
24	18035_3	Nex-cKO-Pos	NA	NA	P4-P5	P21	MOs	Secondary motor area	right	red	[255 0 0]	3
25	18035_8	Nex-cKO-Pos	NA	NA	P4-P5	P21	MOs	Secondary motor area	right	red	[255 0 0]	4
26	19423_4	Nex-cKO-Pos	NA	NA	P4-P5	P21	MOp-MOs	Primary and secondary motor area	right	red	[255 0 0]	5
27	19423_6	Nex-cKO-Pos	NA	NA	P4-P5	P21	SSp	Primary somatosensory area	right	red	[255 0 0]	5
28	11643_17	Nex-cKO-Pos	NA	NA	P4-P5	P21	MOs	Secondary motor area	right	red	[255 0 0]	3
29	19423_7	Nex-cKO-Pos	NA	NA	P4-P5	P21	SSpul	Primary somatosensory area, upper limb	right	red	[255 0 0]	3
30	11431_7	Nex-cKO-Pos	NA	NA	P4-P5	P21	SSpm	Primary somatosensory area, mouth	right	red	[255 0 0]	2
31	11431_3	Nex-cKO-Pos	NA	NA	P4-P5	P21	SSpu-SSpn-SSpb	Somatosensory Cortex	right	red	[255 0 0]	3
32	11431_1	Nex-cKO-Pos	NA	NA	P4-P5	P21	SSpn	Primary somatosensory area, nose	right	red	[255 0 0]	2
33	11431_4	Nex-cKO-Pos	NA	NA	P4-P5	P21	SSpn-SSpm	Somatosensory Cortex	right	red	[255 0 0]	2

Rat wild type	Rat cortex	https://doi.org/10.25493/W8MG-X2R										
expNumber	expName	genotype	strain	sex	age_inject	age_euthani	injectionArea	regionFull	injection	colorName	RGB	gradient
1	M02_BDA	Sprague-Dawley	NA	male	adult	adult	M1	primary motor area	right	black	[0 0 0]	NA
2	M02_Fr	Sprague-Dawley	NA	male	adult	adult	S1-bf	primary somatosensory area, barrel field	right	red	[255 0 0]	NA
3	M18_BDA	Sprague-Dawley	NA	male	adult	adult	M1	primary motor area	right	black	[0 0 0]	NA
4	M18_Fr	Sprague-Dawley	NA	male	adult	adult	S1-bf	primary somatosensory area, barrel field	right	red	[255 0 0]	NA
5	M20_BDA	Sprague-Dawley	NA	male	adult	adult	S1-bf	primary somatosensory area, barrel field	right	black	[0 0 0]	NA
6	M20_Fr	Sprague-Dawley	NA	male	adult	adult	M1	primary motor area	right	red	[255 0 0]	NA
7	M21_BDA	Sprague-Dawley	NA	male	adult	adult	S1-bf	primary somatosensory area, barrel field	right	black	[0 0 0]	NA
8	M21_Fr	Sprague-Dawley	NA	male	adult	adult	M1	primary motor area	right	red	[255 0 0]	NA
9	M22_BDA	Sprague-Dawley	NA	male	adult	adult	S1-bf	primary somatosensory area, barrel field	right	black	[0 0 0]	NA
10	M22_Fr	Sprague-Dawley	NA	male	adult	adult	M1	primary motor area	right	red	[255 0 0]	NA
11	M23_BDA	Sprague-Dawley	NA	male	adult	adult	S1-bf	primary somatosensory area, barrel field	right	black	[0 0 0]	NA
12	M23_Fr	Sprague-Dawley	NA	male	adult	adult	M1	primary motor area	right	red	[255 0 0]	NA
13	M25_BDA	Sprague-Dawley	NA	male	adult	adult	M1	primary motor area	right	black	[0 0 0]	NA
14	M25_Fr	Sprague-Dawley	NA	male	adult	adult	S1-bf	primary somatosensory area, barrel field	right	red	[255 0 0]	NA
15	M26_BDA	Sprague-Dawley	NA	male	adult	adult	S1-bf	primary somatosensory area, barrel field	right	black	[0 0 0]	NA
16	M26_Fr	Sprague-Dawley	NA	male	adult	adult	M1	primary motor area	right	red	[255 0 0]	NA
17	M27_BDA	Sprague-Dawley	NA	male	adult	adult	S1-bf	primary somatosensory area, barrel field	right	black	[0 0 0]	NA
18	M27_Fr	Sprague-Dawley	NA	male	adult	adult	M1	primary motor area	right	red	[255 0 0]	NA
19	M28_BDA	Sprague-Dawley	NA	male	adult	adult	M1	primary somatosensory area, barrel field	right	black	[0 0 0]	NA
20	M28_Fr	Sprague-Dawley	NA	male	adult	adult	S1-bf	primary motor area	right	red	[255 0 0]	NA
21	SS39_BDA	Sprague-Dawley	NA	male	adult	adult	S1-bf	primary somatosensory area, barrel field	right	black	[0 0 0]	NA
22	SS39_Fr	Sprague-Dawley	NA	male	adult	adult	S2	secondary somateosensory area	right	red	[255 0 0]	NA
23	SS40_BDA	Sprague-Dawley	NA	male	adult	adult	S1-bf	primary somatosensory area, barrel field	right	black	[0 0 0]	NA
24	SS40_Fr	Sprague-Dawley	NA	male	adult	adult	S2	secondary somateosensory area	right	red	[255 0 0]	NA
25	SS42_BDA	Sprague-Dawley	NA	male	adult	adult	S1-bf	primary somatosensory area, barrel field	right	black	[0 0 0]	NA
26	SS42_Fr	Sprague-Dawley	NA	male	adult	adult	S2	secondary somateosensory area	right	red	[255 0 0]	NA
27	SS43_BDA	Sprague-Dawley	NA	male	adult	adult	S1-bf	primary somatosensory area, barrel field	right	black	[0 0 0]	NA
28	SS43_Fr	Sprague-Dawley	NA	male	adult	adult	S2	secondary somateosensory area	right	red	[255 0 0]	NA
29	SS45_BDA	Sprague-Dawley	NA	male	adult	adult	S1-bf	primary somatosensory area, barrel field	right	black	[0 0 0]	NA
30	SS45_Fr	Sprague-Dawley	NA	male	adult	adult	S2	secondary somateosensory area	right	red	[255 0 0]	NA
31	SS46_BDA	Sprague-Dawley	NA	male	adult	adult	S1-bf	primary somatosensory area, barrel field	right	black	[0 0 0]	NA
32	SS46_Fr	Sprague-Dawley	NA	male	adult	adult	S2	secondary somateosensory area	right	red	[255 0 0]	NA
33	SS47_BDA	Sprague-Dawley	NA	male	adult	adult	S1-bf	primary somatosensory area, barrel field	right	black	[0 0 0]	NA
34	SS47_Fr	Sprague-Dawley	NA	male	adult	adult	S2	secondary somateosensory area	right	red	[255 0 0]	NA
35	SS48_BDA	Sprague-Dawley	NA	male	adult	adult	S1-bf	primary somatosensory area, barrel field	right	black	[0 0 0]	NA
36	SS48_Fr	Sprague-Dawley	NA	male	adult	adult	S2	secondary somateosensory area	right	red	[255 0 0]	NA
37	SS49_BDA	Sprague-Dawley	NA	male	adult	adult	S1-bf	primary somatosensory area, barrel field	right	black	[0 0 0]	NA
38	SS49_Fr	Sprague-Dawley	NA	male	adult	adult	S2	secondary somateosensory area	right	red	[255 0 0]	NA
39	SS50_BDA	Sprague-Dawley	NA	male	adult	adult	S1-bf	primary somatosensory area, barrel field	right	black	[0 0 0]	NA
40	SS50_Fr	Sprague-Dawley	NA	male	adult	adult	S2	secondary somateosensory area	right	red	[255 0 0]	NA

Rat wild type	Rat barrel	https://doi.org/10.25493/9TMN-64U										
expNumber	expName	genotype	strain	sex	age_inject	age_euthani	injectionArea	regionFull	injection	colorName	RGB	gradient

1	D40_BDA	Sprague-Dawley	NA	male	adult	adult	S1-bf	primary somatosensory area, barrel field	right	black	[0 0 0]	NA
2	D40_Fr	Sprague-Dawley	NA	male	adult	adult	S1-bf	primary somatosensory area, barrel field	right	red	[255 0 0]	NA
3	D41_BDA	Sprague-Dawley	NA	male	adult	adult	S1-bf	primary somatosensory area, barrel field	right	black	[0 0 0]	NA
4	D41_Fr	Sprague-Dawley	NA	male	adult	adult	S1-bf	primary somatosensory area, barrel field	right	red	[255 0 0]	NA
5	D42_BDA	Sprague-Dawley	NA	male	adult	adult	S1-bf	primary somatosensory area, barrel field	right	black	[0 0 0]	NA
6	D42_Fr	Sprague-Dawley	NA	male	adult	adult	S1-bf	primary somatosensory area, barrel field	right	red	[255 0 0]	NA
7	D43_BDA	Sprague-Dawley	NA	male	adult	adult	S1-bf	primary somatosensory area, barrel field	right	black	[0 0 0]	NA
8	D43_Fr	Sprague-Dawley	NA	male	adult	adult	S1-bf	primary somatosensory area, barrel field	right	red	[255 0 0]	NA
9	D44_BDA	Sprague-Dawley	NA	male	adult	adult	S1-bf	primary somatosensory area, barrel field	right	black	[0 0 0]	NA
10	D44_Fr	Sprague-Dawley	NA	male	adult	adult	S1-bf	primary somatosensory area, barrel field	right	red	[255 0 0]	NA
11	D45_BDA	Sprague-Dawley	NA	male	adult	adult	S1-bf	primary somatosensory area, barrel field	right	black	[0 0 0]	NA
12	D45_Fr	Sprague-Dawley	NA	male	adult	adult	S1-bf	primary somatosensory area, barrel field	right	red	[255 0 0]	NA
13	D46_BDA	Sprague-Dawley	NA	male	adult	adult	S1-bf	primary somatosensory area, barrel field	right	black	[0 0 0]	NA
14	D46_Fr	Sprague-Dawley	NA	male	adult	adult	S1-bf	primary somatosensory area, barrel field	right	red	[255 0 0]	NA
15	D48_BDA	Sprague-Dawley	NA	male	adult	adult	S1-bf	primary somatosensory area, barrel field	right	black	[0 0 0]	NA
16	D48_Fr	Sprague-Dawley	NA	male	adult	adult	S1-bf	primary somatosensory area, barrel field	right	red	[255 0 0]	NA
17	D49_BDA	Sprague-Dawley	NA	male	adult	adult	S1-bf	primary somatosensory area, barrel field	right	black	[0 0 0]	NA
18	D49_Fr	Sprague-Dawley	NA	male	adult	adult	S1-bf	primary somatosensory area, barrel field	right	red	[255 0 0]	NA
19	D51_BDA	Sprague-Dawley	NA	male	adult	adult	S1-bf	primary somatosensory area, barrel field	right	black	[0 0 0]	NA
20	D51_Fr	Sprague-Dawley	NA	male	adult	adult	S1-bf	primary somatosensory area, barrel field	right	red	[255 0 0]	NA
21	D52_BDA	Sprague-Dawley	NA	male	adult	adult	S1-bf	primary somatosensory area, barrel field	right	black	[0 0 0]	NA
22	D52_Fr	Sprague-Dawley	NA	male	adult	adult	S1-bf	primary somatosensory area, barrel field	right	red	[255 0 0]	NA
23	D53_BDA	Sprague-Dawley	NA	male	adult	adult	S1-bf	primary somatosensory area, barrel field	right	black	[0 0 0]	NA
24	D53_Fr	Sprague-Dawley	NA	male	adult	adult	S1-bf	primary somatosensory area, barrel field	right	red	[255 0 0]	NA
25	D55_BDA	Sprague-Dawley	NA	male	adult	adult	S1-bf	primary somatosensory area, barrel field	right	black	[0 0 0]	NA
26	D55_Fr	Sprague-Dawley	NA	male	adult	adult	S1-bf	primary somatosensory area, barrel field	right	red	[255 0 0]	NA

Rat wild type	Rat cortex	https://doi.org/10.25493/TH1N-V8P										
**expNumber**	**expName**	**genotype**	**strain**	**sex**	**age_inject**	**age_euthani**	**injectionArea**	**regionFull**	**injection**	**colorName**	**RGB**	**gradient**
1	R401_Pha	wistar	NA	female	adult	adult	S1-fl	Primary somatosensory area, forelimb represe	left	black	[0 0 0]	3
2	R402_BDA	wistar	NA	male	adult	adult	M2	Secondary motor area	left	black	[0 0 0]	3
3	R403_BDA	wistar	NA	male	adult	adult	M2	Secondary motor area	left	black	[0 0 0]	5
4	R405_FG	wistar	NA	female	adult	adult	S1-hl	Primary somatosensory area, hindlimb represe	left	yellow	[255 255 0]	4
5	R405_Fr	wistar	NA	female	adult	adult	S1-hl	Primary somatosensory area, hindlimb represe	left	red	[255 0 0]	4
6	R406_FB	Sprague-Dawley	NA	female	adult	adult	M1	Primary motor area	left	blue	[0 0 255]	2
7	R406_Fe	Sprague-Dawley	NA	female	adult	adult	S1-f	Primary somatosensory area, face representat	left	green	[0 127 0]	2
8	R406_FG	Sprague-Dawley	NA	female	adult	adult	M1	Primary motor area	left	yellow	[255 255 0]	2
9	R406_Fr	Sprague-Dawley	NA	female	adult	adult	S1-f	Primary somatosensory area, face representat	left	red	[255 0 0]	2
10	R407_Fe	Sprague-Dawley	NA	female	adult	adult	S1-fl / S1-hl	Primary somatosensory area, forelimb represe	left	green	[0 127 0]	3
11	R407_Fr	Sprague-Dawley	NA	female	adult	adult	S1-fl	Primary somatosensory area, forelimb represe	left	red	[255 0 0]	3
12	R409_Fr	Sprague-Dawley	NA	female	adult	adult	M2	Secondary motor area	left	red	[255 0 0]	4
13	R410_Fr	Sprague-Dawley	NA	female	adult	adult	M2	Secondary motor area	left	red	[255 0 0]	3
14	R411_BDA	Sprague-Dawley	NA	female	adult	adult	V1	Primary visual area	left	black	[0 0 0]	5
15	R411_Fe	Sprague-Dawley	NA	female	adult	adult	V1	Primary visual area	left	green	[0 127 0]	4
16	R411_Fr	Sprague-Dawley	NA	female	adult	adult	V1	Primary visual area	left	red	[255 0 0]	5
17	R412_BDA	Sprague-Dawley	NA	female	adult	adult	V1	Primary visual area	left	black	[0 0 0]	6
18	R412_Fe	Sprague-Dawley	NA	female	adult	adult	V2M	Secondary visual area, medial part	left	green	[0 127 0]	6

19	R412_Fr	Sprague-Dawley	NA	female	adult	adult	V1	Primary visual area	left	red	[255 0 0]	6
20	R413_BDA	Sprague-Dawley	NA	female	adult	adult	V1	Primary visual area	left	black	[0 0 0]	4
21	R413_Fe	Sprague-Dawley	NA	female	adult	adult	V2L	Primary visual area	left	green	[0 127 0]	3
22	R413_Fr	Sprague-Dawley	NA	female	adult	adult	V1	Primary visual area	left	red	[255 0 0]	5

**Rat wild type**	**Rat SS**	https://doi.org/10.25493/ZSZ9-3NN										
**expNumber**	**expName**	**genotype**	**strain**	**sex**	**age_inject**	**age_euthani**	**injectionArea**	**regionFull**	**injection_**	**colorName**	**RGB**	**gradient**
1	R101_WGA	Sprague-Dawley	NA	female	adult	adult	S1	Primary somatosensory area	right	black	[0 0 0]	
2	R102_BDA	Sprague-Dawley	NA	female	adult	adult	S1	Primary somatosensory area	right	black	[0 0 0]	
3	R103_WGA	Sprague-Dawley	NA	female	adult	adult	S1-f	Primary somatosensory area, face representat	right	black	[0 0 0]	
4	R105_WGA	Sprague-Dawley	NA	female	adult	adult	S1-f	Primary somatosensory area, face representat	right	black	[0 0 0]	
5	R106_Pha	Sprague-Dawley	NA	female	adult	adult	S1-f	Primary somatosensory area, face representat	right	black	[0 0 0]	
6	R109_Pha	Sprague-Dawley	NA	female	adult	adult	S1-f	Primary somatosensory area, face representat	right	black	[0 0 0]	1
7	R113_BDA	Sprague-Dawley	NA	female	adult	adult	S1-f	Primary somatosensory area, face representat	right	black	[0 0 0]	1
8	R115_BDA	Sprague-Dawley	NA	female	adult	adult	S1-fl	Primary somatosensory area, forelimb represe	right	black	[0 0 0]	
9	R117_BDA	Sprague-Dawley	NA	female	adult	adult	S1-tr	Primary somatosensory area, trunk representa	right	black	[0 0 0]	
10	R118_BDA	Sprague-Dawley	NA	female	adult	adult	S1-tr	Primary somatosensory area, trunk representa	right	black	[0 0 0]	
11	R119_BDA	Sprague-Dawley	NA	female	adult	adult	S1-tr	Primary somatosensory area, trunk representa	right	black	[0 0 0]	
12	R120_BDA	Sprague-Dawley	NA	female	adult	adult	S1-tr	Primary somatosensory area, trunk representa	right	black	[0 0 0]	
13	R121_BDA	Sprague-Dawley	NA	female	adult	adult	S1-tr	Primary somatosensory area, trunk representa	right	black	[0 0 0]	
14	R121_Fr	Sprague-Dawley	NA	female	adult	adult	S1-fl	Primary somatosensory area, forelimb represe	right	red	[255 0 0]	
15	R123_BDA	Sprague-Dawley	NA	female	adult	adult	S1-hl	Primary somatosensory area, hindlimb represe	right	black	[0 0 0]	
16	R124_BDA	Sprague-Dawley	NA	female	adult	adult	S1-hl	Primary somatosensory area, hindlimb represe	right	black	[0 0 0]	
